# THE 6-MINUTE WALK TEST AND OTHER CLINICAL ENDPOINTS IN DUCHENNE MUSCULAR DYSTROPHY: RELIABILITY, CONCURRENT VALIDITY, AND MINIMAL CLINICALLY IMPORTANT DIFFERENCES FROM A MULTICENTER STUDY

**DOI:** 10.1002/mus.23905

**Published:** 2013-07-17

**Authors:** Craig M McDonald, Erik K Henricson, R Ted Abresch, Julaine Florence, Michelle Eagle, Eduard Gappmaier, Allan M Glanzman, for the PTC124-GD-007-DMD STUDY GROUP, Robert Spiegel, Jay Barth, Gary Elfring, Allen Reha, Stuart W Peltz

**Affiliations:** 1 Department of Physical Medicine and Rehabilitation, Neuromuscular Medicine and Rehabilitation Research Center, University of California Davis School of MedicineDavis, California, 95817, USA; 2 Department of Neurology, Washington University School of MedicineSt. Louis, Missouri, USA; 3 Institute of Genetic Medicine, Newcastle UniversityNewcastle upon Tyne, UK; 4 Department of Physical Therapy, University of Utah School of MedicineSalt Lake City, Utah, USA; 5 Department of Physical Therapy, Children's Hospital of PhiladelphiaPhiladelphia, Pennsylvania, USA; 6 PTC Therapeutics, South PlainfieldNew Jersey, USA

**Keywords:** 6-minute walk test, ambulation, Duchenne muscular dystrophy, energy expenditure index, muscular dystrophy, myometry, natural history, PedsQL, timed function test

## Abstract

Introduction: An international clinical trial enrolled 174 ambulatory males ≥5 years old with nonsense mutation Duchenne muscular dystrophy (nmDMD). Pretreatment data provide insight into reliability, concurrent validity, and minimal clinically important differences (MCIDs) of the 6-minute walk test (6MWT) and other endpoints. Methods: Screening and baseline evaluations included the 6-minute walk distance (6MWD), timed function tests (TFTs), quantitative strength by myometry, the PedsQL, heart rate–determined energy expenditure index, and other exploratory endpoints. Results: The 6MWT proved feasible and reliable in a multicenter context. Concurrent validity with other endpoints was excellent. The MCID for 6MWD was 28.5 and 31.7 meters based on 2 statistical distribution methods. Conclusions: The ratio of MCID to baseline mean is lower for 6MWD than for other endpoints. The 6MWD is an optimal primary endpoint for Duchenne muscular dystrophy (DMD) clinical trials that are focused therapeutically on preservation of ambulation and slowing of disease progression. *Muscle Nerve* **48**: 357–368, 2013

Duchenne muscular dystrophy (DMD) is a disabling and life-threatening X-linked genetic disorder caused by defects in the gene for dystrophin, a protein that stabilizes muscle cell membranes.^1^ DMD is characterized by complete loss of dystrophin and is the most common neuromuscular disease of childhood. It affects 1 in 3800–6300 males, and there are an estimated 15,000 patients with the disease in the USA.^2^,^3^ There is no approved therapy that addresses the underlying cause of DMD.^4^,^5^ In ∽13% of boys with nonsense mutation DMD (nmDMD, which represents ∽1700 boys in the USA and ∽2400 boys in Europe), the causative defect in the dystrophin gene is a nonsense mutation that truncates dystrophin protein production by introducing a premature stop codon into dystrophin mRNA.^6^–^8^

## THE NEED FOR CLINICALLY MEANINGFUL ENDPOINTS IN DMD

Given that several novel approaches to treatment of DMD have shown promise in preclinical and/or proof-of-concept clinical studies,^9^–^12^ the research community has faced the need to identify and develop clinically meaningful outcome measures for use in pivotal therapeutic trials. In boys with DMD, walking abnormalities are a major disease manifestation that has great importance to patients and families. Ambulation has been noted to be a prerequisite for normal physical functioning in humans^13^; the major goal of medical and physical therapy intervention during the ambulatory phase of DMD is to maintain ambulation for as long as possible.^4^,^5^,^14^,^15^ Given that ambulatory compromise is a key component of the DMD disease process and that ambulation measures the function of multiple muscle groups as well as cardiovascular activity, ambulation-related outcome measures are the most relevant endpoints in DMD patients who are still able to walk. Typically, evaluation of ambulation in DMD features short-term assessments, such as the 10-meter run/walk,^14^,^16^ which measure transient peak activities. The 10-m run/walk test is well accepted and commonly employed in assessing disease progression, but it does not measure endurance, a crucial aspect of ambulatory functioning.

## DEVELOPMENT OF THE 6-MINUTE WALK TEST IN DMD

The 6-minute walk test (6MWT), a well-established outcome measure in a variety of diseases. It is accurate, reproducible, simple to administer, and well tolerated.^17^ It was originally developed as an integrated global assessment of cardiac, respiratory, circulatory, and muscular capacity.^17^ More recently, it has been used to evaluate functional capacity in neuromuscular diseases^18^–^25^ and has been the basis for regulatory registration of several drugs.^19^,^21^,^24^,^25^ Importantly, the 6MWT assesses function and endurance, which are important aspects of DMD patients' disease status. The 6MWT is a robust assessment tool for use in clinical trials given its ability to quantitatively evaluate ambulation in a controlled environment.

In advance of this study, the American Thoracic Society (ATS) version of the 6MWT was modified specifically for DMD.^26^ Also, an orientation video was developed to assist the pediatric subjects (some of whom have cognitive delay) in their understanding of the nature and expectations of the test. In an earlier short-term study, we reported that the modified 6MWT is feasible, safe, and reliable in boys with DMD who have not yet transitioned to full-time wheelchair use.^26^,^27^ We also documented that they have markedly compromised ambulation relative to healthy boys and correlated 6-minute walk distance (6MWD) with age, anthropometric characteristics, and measures, which change with disease progression, including stride length and cadence.^26^,^27^

In a follow-up longitudinal study,^27^ we documented that changes in 6MWD depended on stride length and age; improvements in 6MWD usually occurred up to 7 years of age in both healthy subjects and patients with DMD. However, the 6MWD of older DMD subjects worsened, whereas the 6MWD of older healthy subjects tended to either increase or remain stable. Subsequent studies have demonstrated that the 6MWD correlates with other clinical endpoints in DMD, such as timed function tests and the North Star Ambulatory Assessment (NSAA).^28^,^29^

## MINIMAL CLINICALLY IMPORTANT DIFFERENCE OF ENDPOINTS

Interpretation of functional changes in walk tests can guide clinical management and be primary endpoints in interventional studies. It thus is important to determine whether a change in function is clinically relevant. Data from this study will allow us to determine quantitatively the minimal clinically important difference (MCID) for the test. The MCID is a concept defined as “the smallest difference in score in the domain of interest which patients perceive as beneficial and which would mandate, in the absence of troublesome side effects and excessive cost, a change in patient management.”^30^ The MCID is different from the minimal detectable change, which indicates the amount of change required to exceed measurement variability.^31^,^32^ When interpreting clinical measures, it is important to consider that, although small changes may be significant statistically, they may not be relevant clinically.^31^,^33^ Numerous methods to derive the MCID have been described.^31^,^32^,^34^–^44^ These include anchor-based methods,^31^,^36^,^39^,^40^ which compare a patient's change score with another measure of clinically relevant change, and distribution-based methods,^36^,^41^–^43^ such as the standard error of measurement (SEM), effect size, and one third the standard deviation (SD) at baseline, which are built on the statistical distribution and psychometric properties of the measure in a population. Anchor-based and distribution-based methods are seen as complementary approaches for MCID determination.^44^

## AIMS

There are 3 aims in this report: (1) to assess safety and feasibility of the 6MWT in a large multicenter context; (2) to assess the reproducibility and concurrent validity of the 6MWT results in comparison with other commonly used clinical endpoints utilizing pretreatment results from the international multicenter, randomized, placebo-controlled study of ataluren in ambulatory boys with nmDMD (PTC124-GD-007-DMD, Study 007); and (3) to estimate the MCID using distribution-based methods. These analyses lay the groundwork for longitudinal studies of the 6MWD in DMD.

## METHODS

### Participants

The pretreatment data under evaluation were derived from Study 007; this study enrolled males ≥5 years old at 37 sites in 11 countries (Australia, Belgium, Canada, France, Germany, Israel, Italy, Spain, Sweden, UK, and USA). All patients had phenotypic evidence of dystrophinopathy; had a nonsense mutation in the dystrophin gene as determined by gene sequencing; and walked ≥75 m unassisted during a 6MWT at screening. There was an inclusion criterion of phenotypic evidence of more severe dystrophinopathy based on the onset of characteristic clinical symptoms (i.e., proximal muscle weakness, waddling gait, and Gower maneuver) by 9 years of age, an elevated serum creatine kinase (CK), and ongoing difficulty with ambulation. Patients on systemic glucocorticoids were required to be on a stable dose for 6 months prior to study entry. Institutional review boards/institutional ethics committees and health authorities approved the study protocol. All parents/participants provided signed informed consent/assent before study initiation.

### Overall Study Design and Procedures

Study 007 was a phase 2b, international, multicenter, randomized, double-blind, placebo-controlled, dose-ranging study to evaluate the efficacy and safety of ataluren in ambulatory male patients with nmDMD ≥5 years old. Initial study evaluations (the subject of this report) were performed at screening and baseline separated by up to 6 weeks. Test–retest reliability was determined using the baseline and screening values for all endpoints. Concurrent validity and MCIDs were determined using pretreatment data.

### Subject Disposition and Characteristics

There were 174 randomized patients. All patients screened and randomized were males, ranging in age from 5 to 20 years (Table 1). Approximately 56% of patients were age <9 years, 57% had a baseline 6MWD ≥350 m, and 71% were receiving glucocorticoids. Nonsense mutations were distributed across the 79 exons of the dystrophin gene, with no mutational hotspots identified, and represented all 3 types of premature stop codons.

**Table 1:** Grading used during timed function tests (grades 1–6)

Standing from supine
During the test for standing from a supine position, the method used by the patient was categorized and reported as follows:
1. Unable to stand from supine, even with use of a chair.
2. Assisted Gower―requires furniture for assistance in arising from supine to full upright posture.
3. Full Gower―rolls over, stands up with both hands “climbing up” the legs to above the knees to achieve full upright posture.
4. Half Gower―rolls over, stands up with 1 hand support on lower legs.
5. Rolls to the side and/or stands up with one or both hands on the floor to start to rise.
6. Stands up without rolling over or using hands.
Run/walk 10 m
During the test for running or walking 10 m, the method used by the patient was categorized and reported as follows:
1. Unable to walk independently.
2. Unable to walk independently but can walk with support from a person or with assistive device [full leg calipers (knee-ankle-foot orthoses―KAFOs) or walker].
3. Highly adapted gait, wide-based lordotic gait, cannot increase walking speed.
4. Moderately adapted gait, can pick up speed but cannot run.
5. Able to pick up speed but runs with a double stance phase (i.e., cannot achieve both feet off the ground).
6. Runs and gets both feet off the ground (with no double stance phase).
4-stair climbing
During the test for stair-climbing, the method used by the patient was categorized and reported as follows:
Ascending the stairs:
1. Unable to climb up 4 standard stairs.
2. Climbs 4 standard stairs “marking time” (climbs 1 foot at a time, with both feet on a step before moving to next step), using both arms on one or both handrails.
3. Climbs 4 standard stairs “marking time” (climbs 1 foot at a time, with both feet on a step before moving to next step), using one arm on one handrail.
4. Climbs 4 standard stairs “marking time” (climbs 1 foot at a time, with both feet on a step before moving to next step), not needing handrail.
5. Climbs 4 standard stairs alternating feet, needs handrail for support.
6. Climbs 4 standard stairs alternating feet, not needing handrail support.
4-stair descending
During the test for stair-descending, the method used by the patient was categorized and reported as follows:
Descending the stairs:
1. Unable to descend 4 standard stairs.
2. Descends 4 standard stairs “marking time” (descends 1 foot at a time, with both feet on a step before moving to next step), using both arms on one or both handrails.
3. Descends 4 standard stairs “marking time” (descends 1 foot at a time, with both feet on a step before moving to next step), using one arm on one handrail.
4. Descends 4 standard stairs “marking time” (descends 1 foot at a time, with both feet on a step before moving to next step), not needing handrail.
5. Descends 4 standard stairs alternating feet, needs handrail for support.
6. Descends 4 standard stairs alternating feet, not needing handrail support.

### Outcome Measures

Before the study began, clinical evaluators from each of the 37 participating clinical sites participated in a clinical endpoint training and standardization session to harmonize the testing protocol and logistics across sites. A centralized retraining session was also held ∽1 year after study start. This report focuses on the outcome measures obtained from the patients during screening and baseline.

#### 6-Minute Walk Test/6-Minute Walk Distance

Ambulation was assessed via the 6MWT following standardized procedures as developed at the University of California Davis,^26^ by measuring the 6MWD in meters. The 6MWT for this study included modifications to the method recommended by the ATS for use in adults described in Appendix 2 in the Supporting Information.^17^

#### Timed Function Tests

Timed function tests (TFTs) included time taken to stand from a supine position, time taken to run/walk 10 m, time taken to climb 4 standard-sized stairs, and time taken to descend 4 standard-sized stairs.^45^–^51^ TFTs provide a measure of functional capability in ambulatory patients that is complementary to the 6MWT. The tests are reproducible, simple to administer, and have documented response to therapeutic intervention with steroids.^47^,^48^ A stopwatch was used to time the 10-m run/walk, standing from supine, and 4-stair climb/descend. For standing from supine the velocity was calculated as 1 divided by the time to complete the task. For the total task of climbing 4 standard stairs, velocity was calculated as 1 divided by the time to complete the task. Subjects were given 30 s to complete all tasks. Use of velocities for timed function measures results in a linear pattern of decline that adequately represents the impact of the “zero velocities” of individuals who are unable to perform the evaluation.^52^

#### Timed Function Test Grades

Functional adaptations employed by patients during the TFTs were evaluated and graded by clinical evaluators according to standardized scales developed by one of the investigators (M.E.). Table 1 provides a description of the standardized scales.

#### Myometry

Upper and lower extremity myometry was performed using a hand-held myometer following standardized procedures.^53^–^56^ Muscle groups evaluated included knee flexors, knee extensors, elbow flexors, elbow extensors, and shoulder abductors. Bilateral assessments were done, and 3 measurements (in pounds) were recorded from each muscle group on each side.

#### Health-Related Quality of Life

Health-related quality of life (HRQL) was measured via the Pediatric Quality of Life Inventory (PedsQL).^57^–^60^ The generic core module comprises 23 questions. The PedsQL is available in all languages relevant for this study and was to be completed by both the patient and parent/caregiver. The appropriate age-specific version was completed. It was planned that a patient would be evaluated with the same age-specific form even if during the study an age change made him eligible for a different form. If the patient lacked the ability to complete the PedsQL, the parent/caregiver was still to complete the instrument. If possible, the same parent/caregiver was asked to complete the instrument each time. HRQL was measured by all domains of the PedsQL (Physical, Emotional, Social, and School Functioning domain scores); however, only physical scores are included in this report due to the relative insensitivity of the other domains to disease progression in DMD.^61^

### Data Analysis

Available pretreatment data for all 174 patients from all sites were pooled for analysis of reliability (screening versus baseline performed within 6 weeks), concurrent validity (6MWD in comparison to selected secondary endpoints), and MCID determination for clinical endpoints. For the test–retest analysis in boys with DMD, subjects who had observations at both visits for the parameter of interest were included. Pearson *r* and intraclass correlations (ICCs) for visits 1 and 2 were recorded. For concurrent validity, either the Pearson *r* or Spearman rho (*r*_s_) rank order correlations were calculated. MCID was determined for clinical endpoints using 2 distribution methods: (1) the standard error of measurement method [baseline SD · √(1 − *r*)]; and (2) one third of SD method (baseline SD · ⅓).

#### Percent Predicted 6MWD

To account for maturational effects, including age, height, and associated stride length,^26^,^27^ we calculated a percent predicted 6MWD.^62^,^63^ This prediction equation has been validated in DMD^62^ using the same DMD modified 6MWT protocol as in this study.

#### Energy Expenditure Index

Mean heart rate was measured before (during 5-min rest), during, and for 3 min after the 6MWT with a Polar RS400 heart rate monitor. Using these data, a *post hoc* analysis of energy expenditure index (EEI) was performed. EEI is the active heart rate (beats per minute) minus resting heart rate (beats per minute) divided by walking velocity (meters per minute). Thus, EEI was measured in units of beats per meter. EEI has been documented by Rose and colleagues to be a validated measure of energy expenditure in comparison to oxygen uptake by a metabolic cart in disabled children.^64^–^68^

## RESULTS

### Patient Characteristics

Patient characteristics are shown in Table 2. All patients were males, ranging from 5 to 20 years of age. All 3 premature stop codon types were represented in the study population.

**Table 2:** Patient characteristics (evaluated at screening and baseline)

Characteristics	Baseline (*N* = 174)
Age, years	
Mean (SD)	8.5 (2.6)
Median	8.0
Range	5-20
Race, *n* (%)	
White	157 (90.2)
Black	2 (1.1)
Asian	6 (3.4)
Hispanic	4 (2.3)
Other	5 (2.9)
Body height, cm	
Mean (SD)	125 (13.7)
Median	123
Range	99-173
Body weight, kg	
Mean (SD)	31 (11.5)
Median	27
Range	16-84
Stop codon type, *n* (%)	
UGA	83 (47.7)
UAG	48 (27.6)
UAA	43 (24.7)

### Test–Retest Reliability of Clinical Endpoints

**FIGURE 1:**
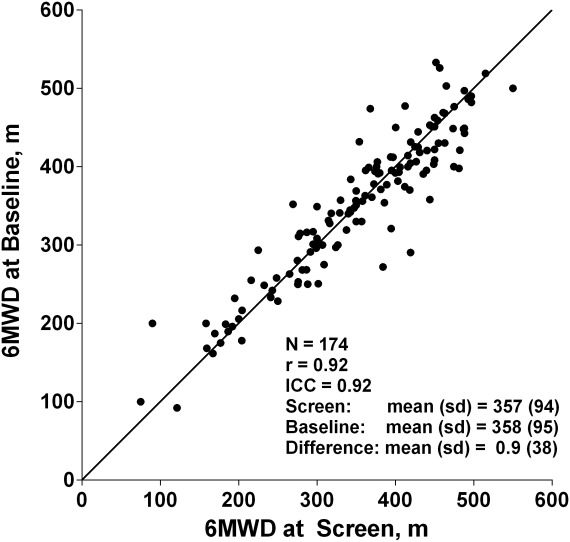
Reliability of the 6-minute walk distance (6MWD) test–retest.

Pretreatment screening and baseline tests were compared for test–retest reliability. The median (range) between-test interval was 42 (0–91) days. Data were available from 174 subjects enrolled at 37 study sites, as shown in Table 3. In general, test–retest reliability for most measures was high. The 6MWD had the highest test–retest reliability of any clinical endpoint (ICC = 0.92), as shown in Figure 1. ICCs for 6MWD, TFTs, and hand-held myometry were all strong (0.72–0.92), as shown in Table 3.

**Table 3:** Test-retest reliability of selected clinical endpoints

Clinical endpoints with timed dimension	ICC	Pearson *r*
6MWD	0.92	0.92
10-m run/walk	0.85	0.87
4-stair climb	0.91	0.91
Stair descend	0.83	0.83
Supine to stand	0.87	0.87

Myometry	ICC	Pearson *r*	ICC	Pearson *r*
	left side	left side	right side	right side
Shoulder abduction	0.76	0.81	0.74	0.78
Elbow flexion	0.82	0.86	0.81	0.95
Elbow extension	0.76	0.75	0.83	0.78
Knee flexion	0.72	0.92	0.77	0.91
Knee extension	0.91	0.86	0.89	0.93

6MWD, 6-minute walk distance; ICC, intraclass correlation coefficient. *P* < 0.0001 for all cases.

### Invalid 6MWT Values due to Musculoskeletal Injuries

Two patients had baseline 6MWD values that deviated markedly from their values at screening (up to 6 weeks earlier) and their first on-treatment values (at week 6). Both patients were documented to have sustained lower limb injuries prior to the baseline test (sprained ankle and right knee injury), which negatively affected their performances at baseline. Their 6MWD values at screening, baseline, and week 6 were 303, 125, and 309 for the first patient and 395, 309, and 481 m for the second patient, respectively. Lower limb injuries are a known source of 6MWD variability. Through a comprehensive analysis, it was verified that these were the only patients in whom the baseline 6MWT results were affected by lower limb injuries.

Considering the strong influence the injuries had on their baseline 6MWD values, it was considered appropriate to declare the baseline test for these patients invalid and to use the screening value as a more accurate reflection of their 6MWD at baseline.

### Concurrent Validity of Clinical Endpoints

For concurrent validity, the following were evaluated:

#### 6MWD vs. Timed Function Tests

**FIGURE 2:**
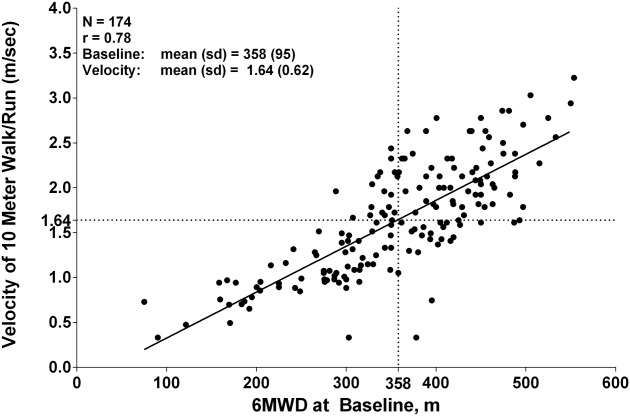
Correlation of velocity during 10-m walk/run vs. 6 minute walk distance (6MWD) at baseline. Note that a mean value of 358 m on 6MWD corresponds to a velocity of 1.64 m/s = 6 s on 10-m run/walk.

Interpretation of time values (in seconds) obtained from timed function testing is limited to those patients who are able to complete the testing, thus creating results biased in favor of more functional individuals. As a result, time scores (in seconds) were converted into velocities. Table 4a shows correlations between 6MWD and velocities for TFTs at baseline (using Pearson *r*). Table 4b shows Spearman rank correlations between method of TFT and the respective time to perform the test. Figure 2 shows the relationship between the baseline velocity during the 10-m run/walk test and baseline 6MWD. It should be noted that a mean value of 358 m on the 6MWD corresponds to a velocity of 1.64 m/s on the 10-m run/walk, which is a time of 6 s for this test.

**Table 4. a:** Pearson correlations between 6MWD and velocity for timed function tests

	6MWD	10-m run/walk	4-stair climb	4-stair descend	Supine to stand
		(m/s)	(stairs/s)	(stairs/s)	(1/s)
6MWD	1.0				
10-m run/walk (m/s)	0.78	1.0			
4-stair climb (stairs/s)	0.77	0.85	1.0		
4-stair descend (stairs/s)	0.73	0.69	0.75	1.0	
Supine to stand (1/s)	0.73	0.86	0.82	0.59	1.0

*P* < 0.0001 in all cases. All other comparisons used Pearson *r*. 6MWD, 6-minute walk distance.

#### Timed Function Test Comparisons

As shown in Table 4a, all TFT velocities had a moderate to high correlation with one another and strong correlations with the specific methods for performance of the TFTs.

#### Myometry vs. 6MWD and Timed Function Tests

Table 5 shows the correlations between right and left myometry measures and 6MWDs, TFTs, and methods of timed function. Knee extension strength correlated better with timed function velocities and with timed function grades based on function, whereas knee flexion strength had lower correlations with timed function velocities and methods of timed function.

**Table 4. b:** Correlations between timed function grade and 6MWD and velocity for timed function tests

	6MWD	10-m run/walk	4-stair climb	4-stair descend	Supine to stand

		(m/s)	(stairs/s)	(stairs/s)	(1/s)
10-m run/walk grade	0.63	0.79			
4-stair climb grade	0.73		0.82		
4-stair descend grade	0.70			0.74	
Supine to stand grade	0.65				0.81

*P* < 0.0001 in all cases. Methods of timed function are only correlated with 6MWD and the time function velocity for the same functional task; comparisons were done using Spearman rho rank order correlations (*r*_s_). 6MWD, 6-minute walk distance.

#### 6MWD vs. Knee Extension Strength

**FIGURE 3:**
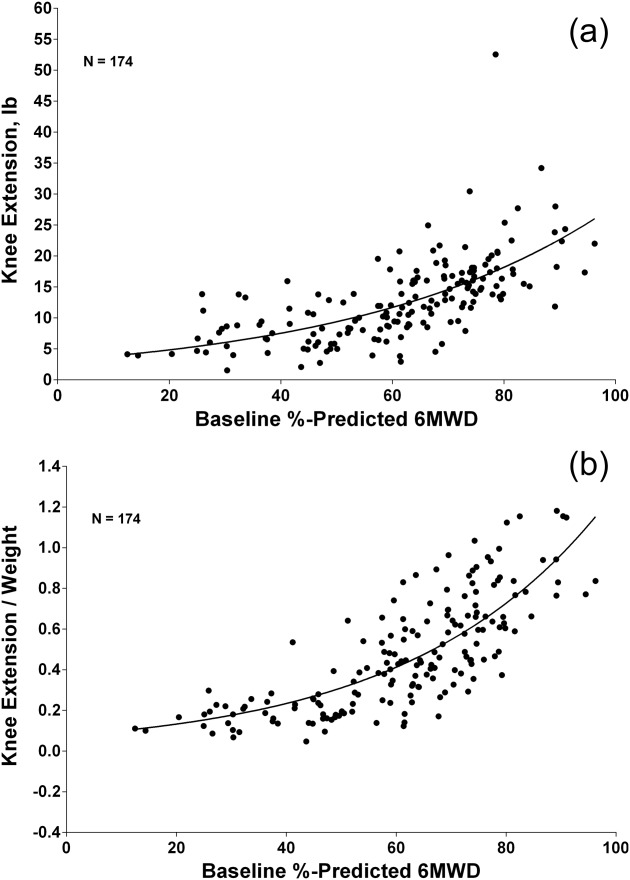
**(a)** Left frame: Correlation of knee extension strength (pounds) with percent predicted 6-minute walk distance (6MWD) at baseline. **(b)** Right frame: Correlation of knee extension strength per kilogram body weight with percent predicted 6MWD at baseline using age- and height-calculated formula.^62^,^63^

Figure 3a and b shows the relationships between knee extension strength and ambulatory function as measured by percent predicted 6MWD. Figure 3a depicts absolute quantitative knee extension strength (in pounds). Figure 3b depicts knee extension strength normalized to body weight. It should be noted that the relationship between these 2 variables (strength and 6MWD) is not linear but logarithmic. In DMD, at reduced 6MWD values below 50–55% predicted (based on age and height), there are substantial declines in ambulatory function as measured by the 6MWD. These declines occur despite relatively small changes in knee extension strength values, which have reached a floor effect.

#### 6MWD vs. Energy Expenditure Index

**FIGURE 4:**
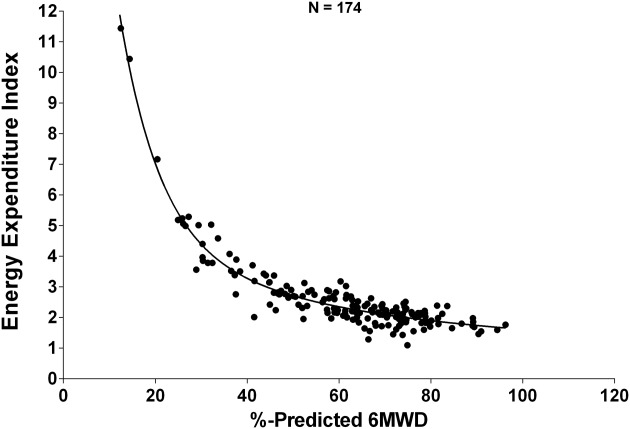
Relationship between energy expenditure index (EEI expressed in units of beats per meter) and percent predicted 6MWD in 174 DMD subjects at baseline.

Figure 4 shows the relationship between heart rate–derived EEI and percent predicted 6MWD in 174 DMD subjects at baseline. The relationship between percent predicted 6MWD and heart rate–derived EEI is logarithmic. When percent predicted 6MWD approaches 50% of control values, there appears to be a precipitous increase in the energy cost of locomotion as measured by the EEI. Thus, the relationship between 6MWD and the EEI is well represented by a negative exponential model.

#### 6MWD vs. PedsQL Physical Function Scale

**FIGURE 5:**
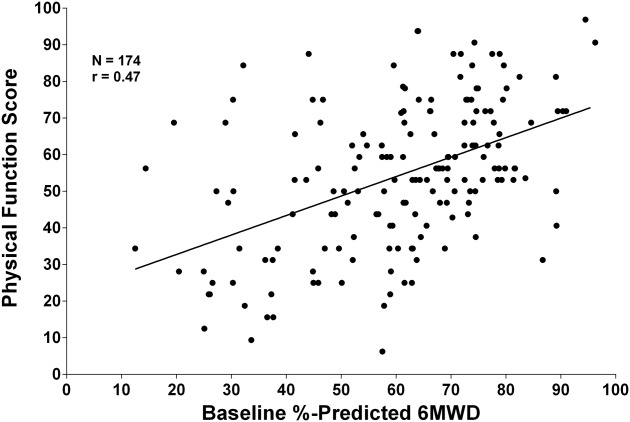
Correlation between 6MWD and PedsQL Physical Function Scale (*n* = 174 DMD evaluated at baseline).

Figure 5 shows the correlation between 6MWD and the PedsQL Physical Function Scale in 174 DMD subjects evaluated at baseline. There was a moderate association between 6MWD and the patient-derived or parent-proxy PedsQL Physical Function Scale (*r* = 0.47).

### Minimal Clinical Important Differences

Distribution-based methods were applied to pretreatment data on all subjects to generate a DMD-specific estimate of the MCID for the following clinical endpoints:

*6MWD*: Based on the standard error of measurement [defined as baseline SD · √(1 − *r*) where *r* is test–retest reliability], the MCID for 6MWD in DMD is estimated to be 28.5 m (Table 6). Based on a definition of one-third of the standard deviation at baseline, the estimated MCID for 6MWD in DMD is 31.7 m (Table 6). These MCID values represent 8.0% and 8.9% of the mean baseline 6MWD.

**Table 9:** MCID for 6MWD in pulmonary and coronary diseases

Disease	Method(s)	MCID (m)	Mean	MCID/ mean baseline	Reference
			baseline	6MWD	
			6MWD (m)		
Interstitial pulmonary fibrosis	Criterion referencing^1^	24	392	6.1%	Du Bois *et al*.^74^ (2011)
	Effect size	31		7.9%	
	SEM	45		11.5%	
Parenchymal lung disease	Criterion referencing^2^	29	403	7.2%	Holland *et al*.^75^ (2009)
	SEM	34		8.4%	
COPD	Effect size	29-42	361	8.0-11.6%	Puhan *et al*.^76^ (2008)
	SEM	35		9.7%	
Coronary artery disease	Criterion referencing^‡^	25	490	5.1%	Gremeaux *et al*.^77^ (2011)
	SEM	23	490	4.7%	

6MWD, 6-minute walk distance; COPD, chronic obstructive pulmonary disease; MCID, minimal clinically important difference; SEM, standard error of measurement.

1 Comparison of baseline 6MWD with occurrence of hospitalization or death during subsequent 48-week period.

2 Comparison of change in 6MWD with change in patient-reported perception of clinical status^75^ or walking ability.^77^

**Table 8:** 6MWT results in prior controlled registration studies

Drug	Indication	*N*	Therapy duration (weeks)	Mean baseline 6MWD (m)	Mean 6MWD improvement^1^ (m) (SD)	% Change in 6MWD	Reference
Bosentan	PPH	213	16	335	44 (NA)	13%	Rubin *et al*.^19^ (2002)
Laronidase	MPS I	45	26	344	38 (68)	11%	Wraith *et al*.^24^ (2004)
Idursulfase	MPS II	96	52	395	30 (61)	8%	Muenzer *et al*.^21^ (2006)
Alglucosidase-α	Pompe disease	90	78	327	28 (56)	9%	van der Ploeg *et al*.^25^ (2010)

6MWD, 6-minute walk distance; 6MWT, 6-minute walk test; MPS, mucopolysaccharidosis; NA, not available; PPH, primary pulmonary hypertension; SD, standard deviation.

Indicates difference between active drug and placebo group over the designated duration of therapy.

**Table 6:** Estimates of MCID for 6MWD and other endpoints in DMD based on pretreatment baseline data

Endpoint/method	*N*	Mean	SD	Correlation*	MCID	MCID/mean
6MWD (m)						
Standard error of measurement method (SD • √(1 – r))	174	358	95	0.91	28.5	8.0%
One third of SD method (SD • ⅓)					31.7	8.9%
Supine to stand (s)						
Standard error of measurement method (SD • √(1 – r))	174	11.5	10.8	0.88	3.7	32.2%
One third of SD method (SD • ⅓)					3.6	31.3%
Climb 4 stairs (s)						
Standard error of measurement method (SD • √(1 – r))	174	6.9	6.6	0.90	2.1	30.4%
One third of SD method (SD • ⅓)					2.2	31.9%
Run/walk 10 m (s)						
Standard error of measurement method (SD * √(1 – r))	174	7.4	4.3	0.71	2.3	31.1%
One third of SD method (SD • ⅓)					1.4	18.9%
Knee extension strength by myometry (lbs.)						
Standard error of measurement method (SD * √(1 – r))	174	13.4	7.1	0.91	2.1	15.7%
One third of SD method (SD • ⅓)					2.4	17.9%

6MWD, 6-minute walk distance; MCID, minimal clinically important difference; SD, standard deviation.

* Based on test-retest reliability at screening and baseline visits ∼6 weeks apart.^47^,^49^

**Table 6:** Correlations between myometry and 6MWD, velocity of timed function, and timed function grade

	Right knee extension	Left knee extension	Right knee flexion	Left knee flexion
	
Pearson correlation				
6MWD (m)	0.64	0.68	0.38	0.42
10-m run/walk (m/s)	0.70	0.69	0.33	0.34
4-stair climb (stair/s)	0.74	0.73	0.37	0.36
4-stair descend (stair/s)	0.58	0.58	0.41	0.43
Supine to stand (1/s)	0.70	0.67	0.28	0.27*
Spearman correlation				
10-m run/walk grade	0.62	0.60	0.17*	0.21*
4-stair climb grade	0.73	0.69	0.33	0.31
4-stair descend grade	0.60	0.61	0.36	0.33
Supine to stand grade	0.66	0.65	0.21*	0.21*

Comparisons with method of timed function use Spearman rho rank order correlations (*r*_s_); all other comparisons done using Pearson *r*. 6MWD, 6-minute walk distance.

*P* < 0.0001 except where noted (NS); *P < 0.05 ^†^*P* < 0.01 and ^‡^*P* < 0.001.

*TFT*: Using similar distribution-based methods for all baseline data for time to stand from supine, time to climb 4 stairs, and time to run/walk 10 m (Table 6), the MCID values represent 18.9–33.9% of the mean baseline TFT values.

*Knee extension*: Using these same distribution-based methods, Table 6 shows the MCID values for knee extension strength by hand-held myometry to be 15.7% and 17.9% of mean baseline knee extension strength.

## DISCUSSION

The findings from this report reflect an evaluation of the largest data set collected to date in a multicenter context for determination of reliability and concurrent validity of 6MWD and other clinical endpoints in DMD. In addition, the investigation has addressed the distribution-based MCID for commonly employed endpoints, including TFTs and quantitative knee extension strength measures, as well as the 6MWD, which is the most common primary endpoint for ambulatory DMD clinical trials.

### Test–Retest Reliability of Measures

The 6MWT showed the highest test–retest reliability of any endpoint used in the study. TFTs, such as the 10-m run/walk test, are easy to administer and conveniently applied in a clinical setting. However, these tests have inherent disadvantages for clinical trials, including slightly reduced test–retest reliability relative to the 6MWT. Advantages of the TFTs as secondary endpoints for DMD trials include previous steroid-naive natural history data^16^ and contemporary long-term natural history data available through the Cooperative International Neuromuscular Research Group (CINRG) Duchenne Natural History Study,^52^ relating the measures to loss of ambulation, the ease of utilizing the measures in the clinic in everyday clinical practice, and their potential inclusion as core measures for registries. Approximately 20% of patients, however, were unable to perform stand from supine at study entry.

To maximize reproducibility, the 6MWT should not be performed in the setting of an acute condition (e.g., musculoskeletal injury) that affects walking ability. In addition, future inclusion criteria can reduce variability by requiring that screening and baseline 6MWD values be within a certain percentage of one another (e.g., 20%).

### Concurrent Validity of Clinical Endpoints

With regard to concurrent validity, the 6MWD was shown to be associated with other measures of disease progression in DMD, but in general it showed closer correlation with TFTs when compared with quantitative strength measures. Time to climb 4 stairs was the timed function measure most highly correlated with knee extension strength, so this may be a particularly useful secondary endpoint for DMD trials. Concurrent validity between the NSAA and 6MWD has been demonstrated previously.^28^,^29^ In this multicenter study the 6MWD correlated highly with the graded methods of performing TFTs, which are evaluator-derived DMD-specific measures of disease progression analogous to several components of the NSAA.

Myometry has been found previously to be less sensitive to changes in disease status than TFTs in ambulatory DMD boys.^69^ Our study has shown that the 6MWT has obvious advantages over quantitative strength measures as an endpoint in DMD due to its sensitivity to detect change in children who have a decline in ambulatory function. In DMD, at reduced 6MWD values below 50–55% predicted (based on age and height), there are significant continued declines in ambulatory function as measured by the 6MWD that occur despite relatively small changes in knee extension strength values, which appear to have approached a floor effect. An alternative concept is that, once lower extremity strength reaches a critically low threshold value, a more precipitous deterioration in ambulatory function may occur over 12 months with relatively little incremental loss of strength during that time.

Although there is a moderate correlation between 6MWD and the parent-proxy–reported PedsQL Physical Function Scale, the relationship is not as strong as that reported between 6MWD or walking speed and other patient-reported outcomes, such as the Transfers/Basic Mobility scale, Sports Physical Functioning Scale, or Global scale from the POSNA/PODCI Pediatric Outcomes Instrument.^61^,^70^

### 6MWT Is an Integrated Global Measure of Ambulatory Function and Metabolic Efficiency

Short-term assessments in DMD that measure transient peak physical activities, such as 10-m run/walk, do not measure endurance, a crucial aspect of ambulatory functioning. Due to the combination of strength loss and cardiopulmonary involvement, children with DMD experience increases in the energy cost of locomotion (more metabolic energy consumed per distance traveled) with increasing disease progression. Other investigators have validated the use of the heart rate–determined EEI as a measure of energy cost in disabled children.^64^–^68^ Heart rate–determined EEI has been validated previously in DMD using a COSMED portable metabolic cart.^71^ Other studies also showed increased energy cost of locomotion in DMD.^72^ Our study has shown that the 6MWD can be considered a proxy measure for the energy cost of locomotion in DMD. In general, higher EEI is associated with more metabolically inefficient ambulation and more impaired endurance. A recent report demonstrated the 6MWT to also be highly correlated with an assisted 6-minute cycling test,^73^ which is a measure of endurance in both ambulatory and non-ambulatory patients with DMD. The 6MWT is therefore an integrated global measure of ambulatory function that is influenced by decreased lower extremity strength, biomechanical inefficiencies during gait, diminished endurance, and compromised cardiorespiratory status.

### Minimal Clinically Important Differences

MCID is a construct that can be determined by statistical distribution approaches, anchor-based methods with patient-reported outcome measures, and determination of clinically meaningful changes with treatments. Prior to the acquisition of disease-specific data, the study of MCID in other diseases is instructive. As shown in Appendix 3 in the Supporting Information, data from placebo-controlled studies of laronidase for mucopolysaccharidosis type I (MPS I), idursulfase for MPS II, bosentan for primary pulmonary hypertension, and alglucosidase-alpha for Pompe disease support the clinical meaningfulness of a 30-m treatment effect for the 6MWT.^19^,^21^,^24^,^25^ In these studies, differences in mean changes in 6MWD in drug-treated patients versus placebo-treated patients ranged from 28 to 44 m, or 8–13% of baseline 6MWD (Appendix 3 in Supplementary Material). The data from the trials in MPS I, MPS II, and Pompe disease are especially relevant given that patient activity limitations in these diseases and those in DMD result from disease-related impairments in neuromuscular and pulmonary systems.

Subsequent to the initiation of the ataluren Study 007 in 2008, additional research had been conducted to define the MCID for 6MWD across multiple diseases, including interstitial pulmonary fibrosis, coronary artery disease, chronic obstructive pulmonary disease, and parenchymal lung disease.^74^–^77^ In each of these diseases, as in DMD, patients experience disease-related deficits in 6MWD relative to healthy controls. Those studies employed multiple methods to determine the MCID for 6MWD, including distribution-based methods utilizing statistical properties (e.g., effect size, standard error of measurement). In these pulmonary and cardiac diseases, estimates of the MCID for 6MWD ranged from 23 to 45 m (Appendix 3 of Supporting Information).^74^–^77^ These estimates of the MCID for 6MWD correspond to 4.7–11.6% of mean baseline 6MWD.

Distribution-based methods are commonly used for initial MCID determination. In this study, we addressed initially the question of MCID for the 6MWD—a relatively new endpoint in DMD—by applying 2 commonly utilized distribution-based methods for DMD-specific MCID determination. The clinical trials that used the 6MWD in other diseases formed the basis of the *a priori* choice of a 30-m 6MWD treatment effect when originally powering the ataluren trial for ambulatory DMD subjects. In the present study of placebo-treated patients, the MCIDs for 6MWD in DMD (corresponding to 8.0% and 8.9% of the mean baseline 6MWD) are both mid-range values compared with the MCIDs determined for the other diseases. Thus, the statistical distribution data provide support for a 6MWD MCID of 30 m in DMD and a targeted 30-m difference between treatment arm and placebo for DMD therapeutic trials. Future longitudinal investigations of anchor-based approaches to MCID are suggested to validate these initial statistical distribution approaches and compare specific changes in 6MWD with the occurrence of both clinically meaningful milestones and significant changes in patient-reported outcomes. These studies may support a lower MCID for 6MWD than 30 m based on distribution-based methods. In addition, more experience evaluating the 6MWD in DMD patients treated with therapeutic agents known to be efficacious will help refine the determination of the MCID for this endpoint.

In this large, multicenter, international clinical trial, the 6MWT proved to be feasible and highly reliable, and it showed excellent concurrent validity with other commonly used clinical endpoints in DMD such as timed function tests and quantitative strength measures. The 6MWD proved to be a more sensitive clinical endpoint when compared with timed function and quantitative strength measures. Statistical distribution approaches support an MCID of ∽30 m for DMD. The 6MWT is an integrated global measure of ambulatory function that is influenced by lower extremity strength, biomechanical inefficiencies, endurance, and cardiorespiratory status. This study and additional longitudinal natural history data^78^ from the ataluren clinical trial (Study 007) support acceptance of the 6MWT as the primary outcome measure of choice for ambulatory DMD clinical trials.

## Appendix 1

### PTC124-GD-007-DMD Study Group Collaborating Authors

**Australia**

*Murdoch Chidlren's Research Institute, Australia Royal Children's Hospital and University of Melbourne, Parkville, Victoria –* Monique Ryan, MBBS

*Department of Clinical Genetics, Children's Hospital at Westmead, New South Wales, Australia –* Kristi Jones, MBBS

**Belgium**

*Department of Pediatrics and Child Neurology, University Hospital Leuven, Belgium* – Nathalie Goemans, MD

**Canada**

*Department of Pediatrics, Children's Hospital Ontario, University of Western Ontario, London, ON, Canada –* Craig Campbell, MD

*Department of Neurology, Alberta Children's Hospital, University of Calgary, Alberta, Canada –* Jean Mah, MD

*Department of Pediatrics, Children's & Womens Health Center of British Columbial, University of British Columbia, Vancouver, BC, Canada–* Kathryn Selby, B.Sc. MBChB, MRCP, FRCPC

**France**

*Institiut de Myologie, Groupe Hospitalier Pitie-Salpetriere, Paris, France –* Pr. Thomas Voit

*Neurologie Pediatrique, Unite de Medecine Infantile Hopital d'Enfants, Marseilles, France –* Pr. Brigitte Chabrol

*Laboratoire d'Exploracion Fonctionnelles, Centre de Reference Maladies Neuromusculaires Nantez-Angers, CHU de Nantes, France –* Pr. Yann Pereon

**Germany**

*Department of Neuropediatrics, Neuromuscular Centre, University of Essen, Essen, Germany –* Dr. med Ulrike Schara

*Department of Pediatrics and Adolescent Medicine, University Hospital Frieberg, Germany –* Dr. Janberndt Kirschner

**Italy**

*Medicina Moleculare di Malattie Neuromuscolari e Neurodegenerative Dipartimento dei Laboratori Oespedale Pediatrico Bambino Gesu di Roma, Italy –* Prof. Enrico Bertini

*Dipartimento di Sciencze Pediatriche Medico-Chiurgiche e Neuroscienze dello Svilippo U.O. Complessa di Neurospichiatria Infantile Policlinica Universitario A. Gemelli, Rome, Italy –* Prof. Eugenio Mercuri

*Unita Operativa di Neurologia Fondazinone IRCCS, Ospedale Maggiore Policlinico, Milan, Italy –* Prof. Giacomo Comi

**Israel**

*Pediatric Neurology Unit, Hadassah Hebrew University Hospital, Jerusalem, Israel –* Prof. Yoram Nevo

**Spain**

*Departamento de Neurologia, Hospital Universitario La Fe, Valencia, Spain Hospital –* Juan Vilchez, PhD,

*Departamento de Neuropediatria, Hospital Sant Joan de Deu, Barcelona, Spain –* Jaume Colomer, PhD

**Sweden**

*Department of Pediatrics, Queen Sylvia Children's Hospital, University of Gothenburg, Sweden* – Mar Tulinius, MD, PhD,

*Department of Women's and Children's Health, Karolinksa University Hospital, Stockholm, Sweden –* Thomas Sejersen, MD, PhD

**United Kingdom**

*Institute of Genetic Medicine, Newcastle University, Newcastle Upon Tyne Great Britain –* Prof. Katherine Bushby

*Department of Neuroscience and Mental Health, King's College, London, UK King's College, London –* Prof. Francesco Muntoni

*National Hospital for Neurology and Neurosurgery, University College London Hospitals, London –* Dr. Rosaline Christina Mary Quinlivan

**United States**

*Department of Pediatrics and Neurology, Cincinnati Children's Medical Center, OH, USA –* Brenda Wong, MD, MBBS

*Department of Pediatrics, The Children's Hospital of Philadelphia, PA, USA –* Richard S. Finkel, MD

*Departments of Neurology and Pediatrics, University of Utah Medical Center, Salt Lake City, UT, USA –* Jacinda B. Sampson, MD, PhD, Kevin M. Flanigan, MD, Russell Butterfield, MD

*Department of Neurology, University of Minnesota, Minneapolis, MN, USA –* John W. Day, MD, PhD

*Department of Neurology, University of Iowa Children's Hospital, Iowa City, IA, USA –* Katherine Mathews, MD

*Department of Neurology, Children's Hospital Boston, MA, USA* – Basil T. Darras, MD, *Department of Rehabilitation Medicine, The Children's Hospital, University of Colorado, Denver, CO, USA –* Susan D. Apkon, MD

*Department of Neurology, The Children's Hospital, University of Colorado, Denver, CO, USA* –Julie Parsons, MD

*Department of Neurology, University of Kansas Medical Center, Kansas City, KS, USA* – Richard Barohn, MD

*Department of Neurology, Washington University School of Medicine at St. Louis, MO, USA –* Anne Connolly, MD,

*Department of Neurology, Children's Medical Center Dallas, University of Texas Southwestern, Dallas, TX, USA –* Susan Iannaccone, MD

*Department of Neurology, Columbia University Pediatric Neuromuscular Center, Columbia University Medical Center, New York, NY, USA –* Douglas M. Sproule, MD, Petra Kaufman, MD, MSc

*Department of Physical Medicine & Rehabilitation, Neuromuscular Medicine & Rehabilitation Research Center, University of California, Davis –* Jay J. Han, MD, Nanette C. Joyce, DO

*Northwest Florida Clinical Research Group, Gulf Breeze, FL, USA* – J. Ben Renfroe, MD

*Department of Neurology, Shriners Hospital for Children Portland, OR, USA –* Barry S. Russman, MD

*Department of Pediatrics and Division of Cardiology, Duke University, Durham, NC, USA* – Stephanie Burns-Wechsler, MD

*Department of Pathology, University of Iowa, Iowa City, IA, USA –* Steven A Moore, MD, PhD

*Department of Physiology, Unversity of Pennsylvania, PA, USA –* H Lee Sweeney, PhD

*OrthoCare Innovations, Mountlake Terrace, WA*, USA – Kim Coleman, MS

## Abbreviations

6MWD: 6-minute walk distance
6MWT: 6-minute walk test
ATS: American Thoracic Society
CINRG: Cooperative International Neuromuscular Research Group
CK: creatine kinase
DMD: Duchenne muscular dystrophy
EEI: energy expenditure index
HRQL: health-related quality of life
MCID: minimal clinically important differences
MPS: mucopolysaccharidosis
nmDMD: nonsense mutation DMD
NSAA: North Star Ambulatory Assessment
PedsQL: Pediatric Quality of Life Inventory
TFT: timed function test
