# Supplementary material for: THE 6-MINUTE WALK TEST AND OTHER CLINICAL ENDPOINTS IN DUCHENNE MUSCULAR DYSTROPHY: RELIABILITY, CONCURRENT VALIDITY, AND MINIMAL CLINICALLY IMPORTANT DIFFERENCES FROM A MULTICENTER STUDY
Source: Muscle Nerve. 2013 Jul 17;48(3):357–68. doi: 10.1002/mus.23905 (PMC3826053; doi:10.1002/mus.23905)
Supplement: Supplementary file 2 [file mus0048-0357-SD2.docx]

**Appendix 2: 6-minute walk test methodology**

The 6MWT for this study included modifications to the method recommended by ATS for use in adults previously validated in DMD at UC Davis.^17^ An orientation video was developed to assist pediatric subjects (some of whom have cognitive delay) in their understanding of the nature and expectations of the test. Other modifications included continuous verbal encouragement from the testing staff to maintain attention to the task, and a “safety chaser” to walk behind the participant during testing and assist them up in the event of a fall. Patients were not permitted use of any assistive devices (e.g. walker, long leg braces, or short leg braces) during the 6MWT. It was recommended that the 6MWT be performed as the final evaluation on the first day of each 2-day clinic visit. If the patient was unable to cooperate with the 6MWT due to behavioral problems on the first day of the visit, the 6MWT could be performed on the second day of the visit.

A test was to be considered valid if the patient completed the test as intended or discontinued the test solely due to exhaustion. A patient was permitted to rest against the wall during the test. A test was to be considered invalid if the patient: a) failed to follow instructions; b) did not remain on the course for the duration of the test; c) moved in a reverse direction for any part of the test; d) discontinued the test prior to 6 minutes due to noncompliance or reasons other than exhaustion (e.g., injury due to fall, sitting down); or e) for other reasons whereby the test was judged by the clinical evaluator and/or investigator as not indicative of a patient’s true ambulatory ability.

Study sites were instructed via the study manual that if a patient did not have a valid test on the initial attempt or was too exhausted to complete the initial test, repeat testing was to be attempted. Repeat tests were to be performed after a break of at least 1 hour on Day 1 and/or on Day 2 of the clinic visit. All attempts were to be made to obtain a valid test for each clinic visit. Ideally, a repeat test on Day 2 was to be performed at approximately the same time of day as was planned for the Day 1 test. If more than 1 valid test was obtained during a visit, the test with the longest achieved distance was to be used in the analysis.

The 6MWT was performed indoors, along a flat, straight, enclosed, and seldom traveled corridor that was generally at least 8 feet wide and had a hard surface. The test area was marked with a 25-m tape-line. The tape-line was placed in the middle of the corridor and marked at 1-m intervals. A cone was positioned at each end of the course with arrows taped to the floor to indicate the counterclockwise direction and path of movement. A stopwatch was used to time the 6MWT. Six sequentially numbered tape flags (1–6) were used to record minute-split distances on the course, and 5 sequentially lettered tape flags (A–E) were used to record points where falls, if any, occurred.
