# Supplementary material for: THE 6-MINUTE WALK TEST AND OTHER CLINICAL ENDPOINTS IN DUCHENNE MUSCULAR DYSTROPHY: RELIABILITY, CONCURRENT VALIDITY, AND MINIMAL CLINICALLY IMPORTANT DIFFERENCES FROM A MULTICENTER STUDY
Source: Muscle Nerve. 2013 Jul 17;48(3):357–68. doi: 10.1002/mus.23905 (PMC3826053; doi:10.1002/mus.23905)
Supplement: Supplementary file 3 [file mus0048-0357-SD3.docx]

**Appendix 3:**

| **Table a. 6MWT Results in Prior Controlled Registration Studies** | | | | | | | | |
| --- | --- | --- | --- | --- | --- | --- | --- | --- |
| **Drug** | | **Indication** | **N** | **Therapy Duration, weeks** | **Mean Baseline 6MWD, m** | **Mean 6MWD**  **Improvement** ^a^**,  m (SD)** | **% Change in 6MWD** | **Reference** |
| Bosentan | | PPH | 213 | 16 | 335 | 44 (NA) | 13% | Rubin 2002 |
| Laronidase | | MPS I | 45 | 26 | 344 | 38 (68) | 11% | Wraith 2004 |
| Idursulfase | | MPS II | 96 | 52 | 395 | 30 (61) | 8% | Muenzer 2006 |
| Alglucosidase-α | | Pompe disease | 90 | 78 | 327 | 28 (56) | 9% | van der Ploeg 2010 |
| a | Indicates difference between active drug and placebo group over the designated duration of therapy | | | | | | | |
| **Abbreviations:** 6MWD = 6-minute walk distance, 6MWT = 6-minute walk test, MPS = mucopolysaccharidosis, NA = not available, PPH = primary pulmonary hypertension, SD = standard deviation | | | | | | | | |

| **Table b. MCID for 6MWD in Pulmonary and Coronary Diseases** | | | | | | |
| --- | --- | --- | --- | --- | --- | --- |
| **Disease** | | **Method(s)** | **MCID, m** | **Mean Baseline 6MWD, m** | **MCID/ Mean Baseline 6MWD** | **Reference** |
| Interstitial pulmonary fibrosis | | Criterion referencing^a^ | 24 | 392 | 6.1% | Du Bois, Weycker et al. 2011 |
|  |  | Effect size | 31 |  | 7.9% |  |
|  |  | SEM | 45 |  | 11.5% |  |
| Parenchymal lung disease | | Criterion referencing^b^ | 29 | 403 | 7.2% | Holland, Hill  et al. 2009 |
|  |  | SEM | 34 |  | 8.4% |  |
| COPD | | Effect size | 29 to 42 | 361 | 8.0% to 11.6% | Puhan, Mador  et al. 2008 |
|  |  | SEM | 35 |  | 9.7% |  |
| Coronary artery disease | | Criterion referencing^c^ | 25 | 490 | 5.1% | Gremeaux, Troisgros et al 2011 |
|  |  | SEM | 23 | 490 | 4.7% |  |
| a | Comparison of baseline 6MWD with occurrence of hospitalization or death during subsequent 48-week period | | | | | |
| b | Comparison of change in 6MWD with change in patient-reported perception of clinical status (Holland 2009) or walking ability (Gremeaux 2011) | | | | | |
| **Abbreviations:** 6MWD = 6-minute walk distance, COPD = chronic obstructive pulmonary disease, MCID = minimal clinically important difference, SEM = standard error of measurement | | | | | | |
